# Remote Sensing, Crowd Sensing, and Geospatial Technologies for Public Health: An Editorial

**DOI:** 10.3390/ijerph14040405

**Published:** 2017-04-11

**Authors:** Jamal Jokar Arsanjani

**Affiliations:** Geoinformatics Research Group, Department of Planning, Aalborg University Copenhagen, A.C. Meyers Vænge 15, DK-2450 Copenhagen, Denmark; jja@plan.aau.dk

Remote sensing, as well as the recent advancements of crowd sensing, along with novel and recent geospatial technologies, have great potential to explore and understand the relationships between our surroundings—in particular our urban and rural environments and natural spaces—and public health through environmental factors [1,2]. Emerging phenomena including climate change, extreme weather conditions, dynamic and mega cities, air pollution, and dust storms, among others, have significant impacts on human and environmental health [3]. On the one hand, the rising volume of Earth observatories and citizen observatories has provided research scholars with a tremendous amount of data streams in space and time, which are novel, unique, and even freely available; therefore, new research agendas are to be designed to exploit the power of these data [4,5]. On the other hand, recent geospatial technologies, such as novel geocomputational techniques, clustering algorithms, visual analytics, data/information mining approaches, Web 2.0, and collaborative sensing techniques, among others, have presented a wide variety of techniques for exploring these data and discovering latent information about public health [6].

To address these issues, the prime aim of this Special Issue is to present novel sensing and computational techniques in order to better understand public health, developing diverse public health applications, and to explore their underlying implications. Thus, we can move towards securing healthier urban/rural environments and natural spaces. This collection of papers should provide a selection of interesting approaches and methodologies, useful for audiences including researchers, practitioners, and professionals.

In February 2016, this Special Issue was started by announcing widely the call for papers, and inviting leading scholars to contribute. It received a great response by a significant number of scholars. By the deadline for submission in September 2016, a total of 23 submissions were submitted. Through a single-blind review process following standard MDPI review guidelines, we invited at least three expert reviewers to review the manuscripts and comment on the quality, originality, relevance, as well as fit for the Special Issue. This led to 12 of the 23 submitted manuscripts being accepted (almost 52% acceptance rate) for publication and thus were included in the Special Issue.

The Special Issue consists of the following papers: Zhang et al. present a Geographically Weighted Regression (GWR) model using NO_2_, Enhanced Vegetation Index (EVI), Aerosol Optical Depth (AOD) product, and other meteorological parameters to explain the variation of PM_2.5_ across China [7]. They also provide a discussion on the usefulness of their results as a reasonable reference for assessing health impacts as well as examining the effectiveness of emission control strategies being implemented in China. Wu et al. explore the spatial associations between thrombocytopenia syndrome virus (SFTSV) infections and several potential determinants (average temperature, average monthly precipitation and average relative humidity) using a GWR model in order to predict the high-risk areas in China [8]. Their proposed approach is suggested for predicting high-risk areas in the implementation of public health strategies. Wang et al. present a study for estimating long-term urban nocturnal boundary layers (NBLs) using elastic backscatter light detection and ranging (LiDAR) in Wuhan, China [9]. They explore (a) the relationship between NBL height (NBLH) and near-surface parameters to elucidate meteorological processes governing NBL variability; and (b) the influence of NBLH variations in surface particulate matter (PM). Wang et al., using Jiangsu as a case study, investigate the reduction in arable land via a long-term dynamic monitoring scheme for arable land quality [10]. They discuss the results of their optimization scenarios.

Zhang et al. introduce an adapted semi-physical GWR model for real-time estimation of PM_2.5_ mass concentrations at national scale using the Aqua Moderate Resolution Imaging Spectroradiometer (MODIS) Aerosol Optical Depth product for China [11]. They believe that their findings could provide reasonable references for assessing health impacts and offer guidance on air quality management in China. Shi et al. present a study for identifying the uncertainty in a physician’s practice location through spatial analytics, text mining, and visual examination [12]. Their research can have a broader impact on federal and state initiatives and policies to address both insufficiency and maldistribution of a health care workforce in order to improve the accessibility to public health services.

Young et al. [13], using Egypt as a case study, identify areas where co-infection can most likely help target spaces for increased surveillance. Ecological niche modelling using remote sensing data is used for this purpose and they identified environmental, behavioural, and population characteristics of H5N1 and H9N2 niches within Egypt. Ni et al. [14] analyse the relationship between the road network and the healthcare facility distribution network by applying point pattern analysis and correlation analysis. Their results map the spatial distribution of healthcare facilities and their clustered and evenly distributed patterns; and visualize the significant differences across the study area. Their proposed method and findings can help to assess the reasonability of existing healthcare facilities distribution in order to optimize their location.

Sun et al. [15] investigate the concentrations of twelve elements in the topsoil across Beijing using geostatistics and multivariate statistics in order to identify the spatial distribution of characteristics and sources. In addition, the health risk of the heavy metals to humans was evaluated. Their findings reveal a remarkable variation in spatial scale. Finally, health risk assessment for each of the elements is discussed. Wang et al. [16] assess the particle Lidar ratios over Wuhan, China, using a Raman Lidar from July 2013 to May 2015 in order to obtain homogeneous aerosol Lidar ratios near the surface through the Raman method during no-rain nights. Their results indicate that the large Lidar ratio values correspond well with weak winds and strong northerly winds, whereas significantly low Lidar ratio values are associated with prevailing south-westerly and southerly wind. Ebhuoma et al. [17] present a review paper, based on an analysis of 35 peer-reviewed articles, for modelling the spatial and temporal risk of malaria transmission across Sub-Saharan Africa using remote sensing-driven climatic/environmental variables. Their conclusions reveal that the normalized difference vegetation index (NDVI) derived from NOAA-AVHRR and MODIS satellite sensors was most frequently referred to as a statistically-significant variable to model both spatial and temporal malaria transmission. Furthermore, generalized linear models e.g., linear regression, logistic regression and Poisson regression were the most frequently employed methods of statistical analysis in determining malaria transmission predictors.

In conclusion, these papers demonstrate a great deal of contribution from remote sensing data and geospatial technologies combined with in situ observations which were embedded in order to study factors affecting public health across different global regions. Unexpectedly, there was no submission dealing with crowdsourced data in exploring our landscapes and its connection with public health parameters. This selection addresses a broad range of aspects, leveraging on the multidisciplinary vision of health and environmental research. It should be noted that the wealthy and diversified body of tools available within the field of geoinformation technologies will steer this area to become an increasingly present concept in studying issues concerned with public health planning and decision making.
